# Due to Increased Immune Therapies, Are Sensitized Heart Transplant Recipients at Increased Risk for Malignancies?

**DOI:** 10.3389/ti.2026.15593

**Published:** 2026-01-29

**Authors:** Masaki Tsuji, Michelle M. Kittleson, David H. Chang, Evan P. Kransdorf, Andriana P. Nikolova, Lily K. Stern, Mason Lee, Jon A. Kobashigawa

**Affiliations:** 1 Department of Cardiology, Smidt Heart Institute, Cedars-Sinai Medical Center, Los Angeles, CA, United States; 2 Department of Cardiovascular Medicine, Graduate School of Medicine, The University of Tokyo, Tokyo, Japan

**Keywords:** cancer after transplant, desensitization, heart transplant, malignancy, sensitized

Dear Editors,

Sensitization leads to the formation of antibodies to human leukocyte antigens (HLA) [1]. Among sensitized heart transplant (HT) candidates, the waiting time for HT is longer along with the risk of adverse events [2]. Moreover, the presence of HLA antibodies reduces rate of survival and increases the risk of rejection and cardiac allograft vasculopathy [3].

These rejection episodes require increased immunosuppression, which in turn raises concerns about adverse effects such as malignancies. Moreover, desensitization, including intravenous immunoglobulin (IVIG), plasmapheresis, and several anti-humoral agents, is performed for highly sensitized patients for an increase in the chances of a negative crossmatch, expansion of the donor pool, and improvement of post-HT outcomes.

However, the relationship between sensitization and post-transplant malignancies (PTM) has not been well understood. Therefore, we investigated the incidence of PTM and the impact of desensitization for PTM in sensitized HT recipients.

This study design is illustrated in Supplementary Figure S1. We reviewed the records of adult patients who underwent HT between 2010 and 2023 and excluded those with a history of malignancies and missing data. Sensitization was defined as a panel-reactive antibody (PRA) level (either class I or II) of ≥10%. Highly sensitized patients were considered for desensitization therapy. Desensitization included Rituximab, Eculizumab, Bortezomib, Tocilizumab and Obinutuzumab treatment. Our institutional protocol for post-transplant management has been previously described [4, 5].

The primary endpoint of this study was the incidence of PTM diagnosed based on histological evidence. The secondary end point was all-cause mortality. The study participants were followed up until 31 August 2024. Event-free survival analyses were performed using the Kaplan–Meier method and compared using the log-rank test. A Cox proportional hazard model was constructed, adjusting for sensitization status, age, and sex. Nearest-neighbor propensity matching was performed to generate matched cohort. The propensity score model was developed using the following covariates: recipient age, sex, and history of HT. The study protocol was approved by the Institutional Review Board of Cedars-Sinai. Written informed consent was obtained from all patients.

Among the 1,096 patients who underwent HT, 364 were sensitized and 110 were desensitized. The overall mean age was 55.1 ± 12.9 years, and 801 (73.1%) patients were male. The mean follow-up period was 6.4 ± 3.8 years.

The baseline patient characteristics of the sensitized and non-sensitized groups are presented in Supplementary Table S1. The sensitized group was significantly younger, with a higher proportion of female, history of pregnancy, history of blood transfusion, history of HT, and sex mismatch than the non-sensitized group. The mean follow-up period was 6.5 ± 3.9 years in the sensitized group and 6.4 ± 3.8 years in the non-sensitized group (p = 0.68). During the follow-up period, 183 (16.7%) patients developed PTM, with skin cancer being the most common, followed by genitourinary/gynecologic/renal cancers (Supplementary Table S2). Figure 1A shows the difference in freedom from PTM, which was significant between the sensitized and non-sensitized groups (p = 0.041). The 10-year freedom from PTM was 77% in the sensitized group and 69% in the non-sensitized group. However, the all-cause mortality was similar between the two groups (p = 0.68, Figure 1B). In the multivariable Cox analysis, sensitized status was not associated with PTM (Supplementary Table S3).

**FIGURE 1:**
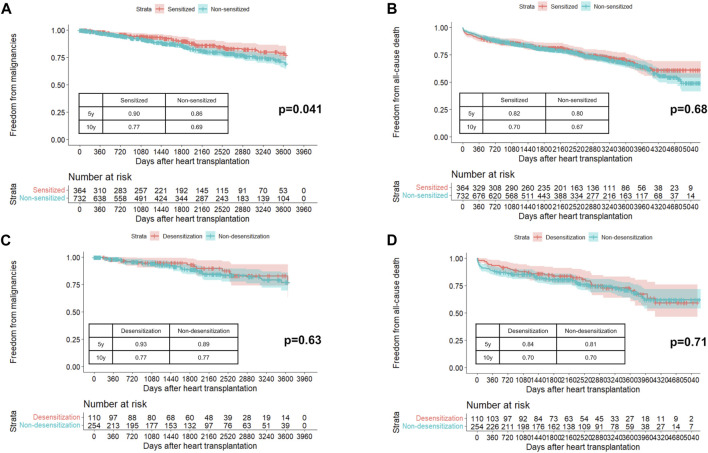
Probability of freedom from post-transplant malignancies **(A)** and all-cause death **(B)** in sensitized and non-sensitized groups. Probability of freedom from post-transplant malignancies **(C)** and all-cause death **(D)** in desensitization and non-desensitization groups in unmatched cohort.

The baseline patient characteristics of the desensitization and non-desensitization groups are presented in Supplementary Table S4. Before propensity score matching, the desensitization group was younger, had a higher proportion of females, and had a higher body mass index. The peak PRA value was significantly higher in the desensitization group than in the non-desensitization group. The most common agent for desensitization was rituximab (56.4%) followed by eculizumab (38.2%). The mean follow-up period was 6.9 ± 3.7 years in the desensitization group and 6.4 ± 3.9 years in the non-desensitization group (p = 0.31). In the unmatched cohort, freedom from PTM (Figure 1C) and all-cause mortality (Figure 1D) were similar between the two groups (p = 0.63 and p = 0.71). After propensity matching with one-to-one pairs, 108 patients in the desensitization group had a higher proportion of multi-organ transplants, whereas age, sex, and body mass index were similar (Supplementary Table S4). In the matched cohort, PTM incidence (Supplementary Figure S2A) and all-cause death (Supplementary Figure S2B) were comparable between the two groups (p = 0.43 and p = 0.58, respectively).

In this study, we identified the following: (1) Sensitized patients were younger, more often female, and had a history of pregnancy, blood transfusion, or HT. (2) The incidence of PTM in sensitized patients was lower than that in non-sensitized patients. (3) Desensitization did not lead to the development of PTM in sensitized patients.

Risk factors for sensitization include prior pregnancy, blood transfusions, infections, presence of homografts/allografts, and use of temporary or durable mechanical circulatory support [3]. Data from the United Network of Organ Sharing dataset for bridge-to-transplant patients show that sensitized patients tend to be younger and female [6]. Conversely, the risk factors for PTM, as identified in several large cohort analyses, include older age at HT, male sex, infection with oncogenic viruses, re-transplantation, and malignancies prior to HT. The risks of sensitization and PTM are inversely related to age and sex. The lower incidence of PTM in sensitized patients in our study might be explained by their younger age and higher proportion of females.

The safety of desensitization agents in terms of PTM risk is not well established, and reports on the association between desensitization and PTM in solid-organ transplants are limited. Bachelet, et al. found no difference in the incidence of PTM between sensitized kidney transplant recipients treated with Rituximab and those who were not [7]. On the contrary, a report from Taiwan showed that patients who underwent desensitization with Rituximab, plasmapheresis and IVIG in kidney transplant had a higher incidence of PTM, particularly urothelial carcinoma [8]. There are no reports on other desensitization agents beside Rituximab nor are there studies in HT population. To the best of our knowledge, our study is the first to evaluate desensitization and PTM in the field of HT and suggests no significant difference in PTM incidence between groups after adjusting for baseline characteristics.

This study has some limitations. First, this was a retrospective, single-center study with a small cohort. Second, IVIG and plasmapheresis were not defined as desensitization. The general categories of desensitization therapy include mechanical removal of antibodies, IVIG, and immunosuppressive agents targeting antibody production; however, this study focused on immunosuppressive agents targeting antibody production. Third, the targets of the humoral immune pathway for each agent used for desensitization were different, and further investigation of the PTM risk associated with each agent is necessary. Fourth, malignancy-related data, including stage and severity, were missing. Finally, oncogenic viral infections were not identified; therefore, their involvement remained unclear.

In conclusion, our findings suggest that neither sensitization nor desensitization therapies were associated with an increased incidence of PTM in this cohort; however, these results should be interpreted cautiously given the potential for residual confounding and the limitations of the retrospective design.

## Data Availability

The raw data supporting the conclusions of this article will be made available by the authors, without undue reservation.
