# Supplementary figures and images for: Due to Increased Immune Therapies, Are Sensitized Heart Transplant Recipients at Increased Risk for Malignancies?
Source: Transpl Int. 2026 Jan 29;39:15593. doi: 10.3389/ti.2026.15593 (PMC12894043; doi:10.3389/ti.2026.15593)

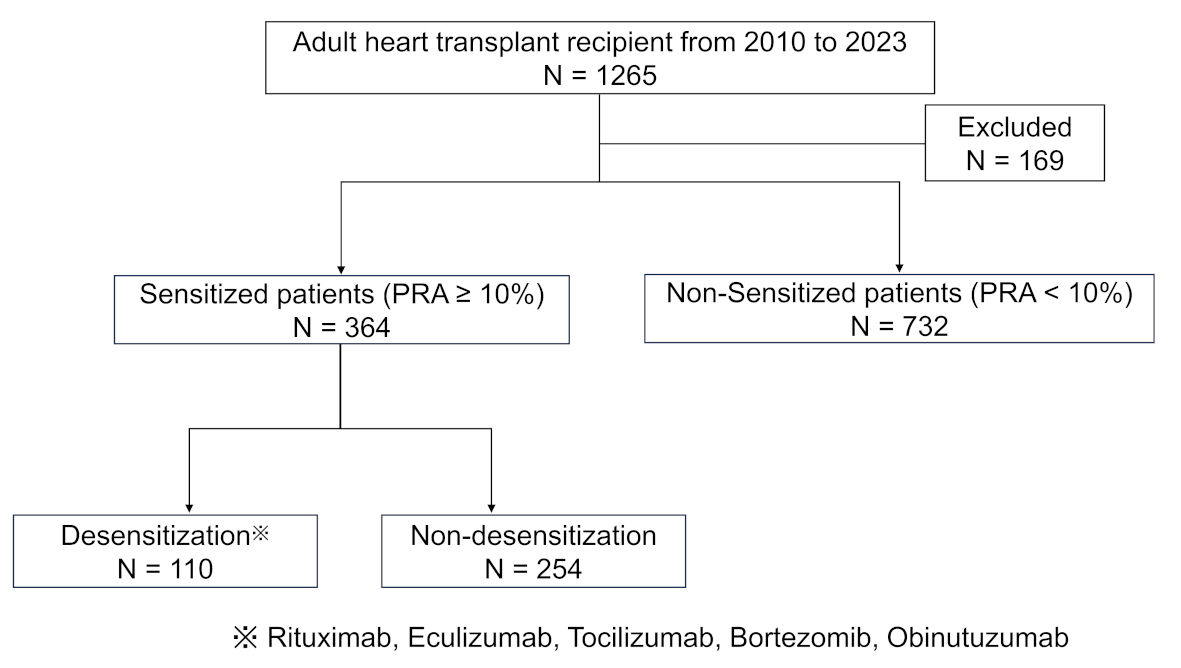

Supplement: Supplementary file 1 [file Image1.tiff]

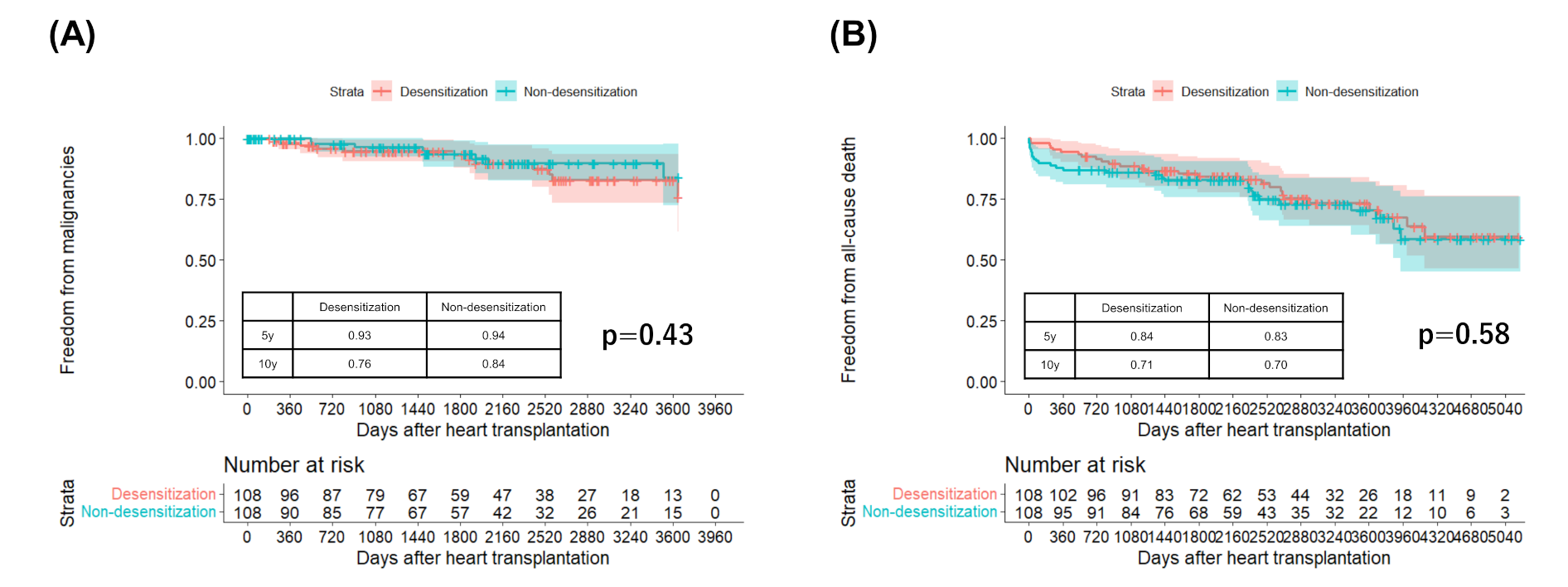

Supplement: Supplementary file 6 [file Image2.tiff]
