# Supplementary material for: Due to Increased Immune Therapies, Are Sensitized Heart Transplant Recipients at Increased Risk for Malignancies?
Source: Transpl Int. 2026 Jan 29;39:15593. doi: 10.3389/ti.2026.15593 (PMC12894043; doi:10.3389/ti.2026.15593)
Supplement: Supplementary file 2 [file Table1.docx]

Table S1. Comparison of baseline and clinical findings in sensitized and non-sensitized groups.

| **Variables** | **Sensitized group**  **(n = 364)** | **Non-sensitized group**  **(n = 732)** | **p value** |
| --- | --- | --- | --- |
| **Recipient profile** |  |  |  |
| Sex, male | 207 (56.9%) | 594 (81.1 %) | **<0.001** |
| Age (years) | 51.9 ± 13.9 | 56.0 ± 12.4 | **<0.001** |
| Ethnicity, Hispanic | 70 (19.2%) | 125 (17.1%) | 0.38 |
| Body mass index (kg/m^2^) | 25.6 ± 4.7 | 25.8 ± 4.6 | 0.64 |
| **Medical history** |  |  |  |
| Hypertension | 223 (61.3%) | 427 (58.3%) | 0.35 |
| Diabetes mellitus | 114 (31.3%) | 266 (36.3%) | 0.10 |
| Pre mechanical circulatory support | 134 (36.8%) | 227 (31.0%) | 0.054 |
| History of blood transfusion | 175 (48.1%) | 220 (30.1%) | **<0.001** |
| History of pregnancy in female | 129/157 (82.2%) | 90/138 (65.2%) | **<0.001** |
| Previous transplant | 44 (12.1%) | 26 (3.6%) | **<0.001** |
| **Donor profile** |  |  |  |
| Donor sex, male | 250 (68.7%) | 522 (71.3%) | 0.37 |
| Donor age (years) | 34.2±11.9 | 35.1±12.4 | 0.26 |
| **Transplant Profile** |  |  |  |
| Peak PRA (%) | 54.1±29.1 | 0.56±1.9 | **<0.001** |
| Ischemic time (min) | 181.8±53.0 | 179.8±52.5 | 0.55 |
| Multi-organ transplant | 55 (15.1%) | 112 (15.3%) | 0.93 |
| Sex mismatch | 101 (27.7%) | 154 (21.0%) | **0.013** |
| Cytomegalovirus mismatch | 71 (19.5%) | 159 (21.7%) | 0.40 |

PRA; panel-reactive antibody.
