# Supplementary material for: Due to Increased Immune Therapies, Are Sensitized Heart Transplant Recipients at Increased Risk for Malignancies?
Source: Transpl Int. 2026 Jan 29;39:15593. doi: 10.3389/ti.2026.15593 (PMC12894043; doi:10.3389/ti.2026.15593)
Supplement: Supplementary file 3 [file Table2.docx]

**Table S2.** Types and frequencies of post-transplant malignancies in sensitized and non-sensitized group.

| Types of malignancies | Sensitized group  (n = 49) | Non-sensitized group  (n = 134) |
| --- | --- | --- |
| Skin | 32 (65.3%) | 94 (70.1%) |
| Genitourinary/Gynecologic/Renal | 8 (16.3%) | 14 (10.4%) |
| Gastrointestinal | 3 (6.1%) | 11 (8.2%) |
| Hematologic | 3 (6.1%) | 11 (8.2%) |
| Lung | 1 (2.0%) | 1 (0.7%) |
| Breast | 1 (2.0%) | 1 (0.7%) |
| Other | 1 (2.0) | 2 (1.5%) |
