# Supplementary material for: Due to Increased Immune Therapies, Are Sensitized Heart Transplant Recipients at Increased Risk for Malignancies?
Source: Transpl Int. 2026 Jan 29;39:15593. doi: 10.3389/ti.2026.15593 (PMC12894043; doi:10.3389/ti.2026.15593)
Supplement: Supplementary file 4 [file Table3.docx]

**Table S3,** Multivariable Cox proportional hazard analysis of parameters predicting post-transplant malignancies.

| **Variables** | **Hazard ratio** | **95% confidence interval** | **p value** |
| --- | --- | --- | --- |
| Sex, male  Age at heart transplant, per year  Sensitization status, Sensitized | 1.70  1.07  0.96 | 1.16-2.50  1.05-1.09  0.68-1.34 | 0.007  <0.001  0.80 |
