# Supplementary material for: Due to Increased Immune Therapies, Are Sensitized Heart Transplant Recipients at Increased Risk for Malignancies?
Source: Transpl Int. 2026 Jan 29;39:15593. doi: 10.3389/ti.2026.15593 (PMC12894043; doi:10.3389/ti.2026.15593)
Supplement: Supplementary file 5 [file Table4.docx]

Table S4. Comparison of baseline and clinical findings in sensitized and non-sensitized groups.

|  | **Unmatched cohort** | |  | **Matched cohort** | |  |
| --- | --- | --- | --- | --- | --- | --- |
| **Variables** | Desensitization group (N=110) | Non-desensitization group (N= 254) | p value | Desensitization group (N=108) | Non-desensitization group (N=108) | p value |
| **Recipient profile** |  |  |  |  |  |  |
| Sex, male | 44 (40.0%) | 163 (64.2%) | **<0.001** | 44 (40.7%) | 51 (47.2%) | 0.34 |
| Age (years) | 49.3 ± 14.2 | 53.1 ± 13.6 | **0.017** | 49.7 ± 14.0 | 49.5 ± 15.1 | 0.93 |
| Ethnicity, Hispanic | 19 (17.3%) | 51 (20.1%) | 0.53 | 19 (17.6%) | 24 (22.2%) | 0.39 |
| Body mass index (kg/m^2^) | 27.9 ± 6.3 | 26.6 ± 5.4 | **0.040** | 24.9 ± 4.4 | 26.1 ± 5.1 | 0.063 |
| Medical history |  |  |  |  |  |  |
| Hypertension | 69 (62.7%) | 154 (60.6%) | 0.71 | 68 (63.0%) | 63 (58.3%) | 0.49 |
| Diabetes mellitus | 29 (26.4%) | 85 (33.5%) | 0.18 | 29 (26.9%) | 31 (28.7%) | 0.76 |
| Pre mechanical circulatory support | 40 (36.4%) | 94 (37.0%) | 0.91 | 39 (36.1%) | 36 (33.3%) | 0.67 |
| History of blood transfusion | 53 (48.2%) | 122 (48.0%) | 0.98 | 52 (48.1%) | 53 (49.1%) | 0.89 |
| History of pregnancy in female | 57/66 (86.4%) | 72/91 (79.1%) | 0.24 | 56/64 (87.5%) | 39/57 (68.4%) | **0.011** |
| Previous transplant | 13 (11.8%) | 31 (12.2%) | 0.92 | 13 (12.0%) | 20 (18.5%) | 0.19 |
| **Donor profile** |  |  |  |  |  |  |
| Donor sex, male | 68 (61.8%) | 182 (71.7%) | 0.063 | 68 (63.0%) | 73 (67.6%) | 0.47 |
| Donor age (years) | 34.1 ± 12.3 | 34.2 ± 11.6 | 0.95 | 34.2 ± 12.4 | 33.0 ± 11.8 | 0.49 |
| **Transplant Profile** |  |  |  |  |  |  |
| Peak PRA (%) | 72.8 ± 29.1 | 46.0 ± 25.1 | **<0.001** | 72.7 ± 29.2 | 51.7 ± 27.0 | **<0.001** |
| Ischemic time (min) | 176.5 ± 49.6 | 184.1 ± 54.3 | 0.21 | 176.7 ± 49.4 | 187.9 ± 46.5 | 0.092 |
| Multi-organ transplant | 13 (11.8%) | 42 (16.5%) | 0.25 | 13 (12.0%) | 25 (23.1%) | **0.032** |
| Sex mismatch | 34 (30.9%) | 67 (26.4%) | 0.38 | 34 (31.5%) | 34 (31.5%) | 0.99 |
| Cytomegalovirus mismatch | 18 (16.4%) | 53 (20.9%) | 0.32 | 17 (15.7%) | 23 (21.3%) | 0.29 |
| **Desensitization** |  |  |  |  |  |  |
| Rituximab | 62 (56.4%) | N/A |  | 61 (56.5%) | N/A |  |
| Eculizumab | 42 (38.2%) | N/A |  | 41 (38.0%) | N/A |  |
| Bortezomib | 39 (35.5%) | N/A |  | 38 (35.2%) | N/A |  |
| Tocilizumab | 23 (20.9%) | N/A |  | 22 (20.4%) | N/A |  |
| Obinutuzumab | 19 (17.3%) | N/A |  | 18 (16.7%) | N/A |  |
